# NO Addition during Gas Oxygenation Reduces Liver and Kidney Injury during Prolonged Cardiopulmonary Bypass

**DOI:** 10.3390/pathophysiology30040037

**Published:** 2023-10-19

**Authors:** Aleksey Maksimovich Radovskiy, Andrey Evgenevich Bautin, Alexander Olegovich Marichev, Victor Vasilyevich Osovskikh, Natalia Yuryevna Semenova, Zoya Evgenyevna Artyukhina, Lada Aleksandrovna Murashova, Vsevolod Alexandrovich Zinserling

**Affiliations:** Almazov National Research Center, Saint-Petersburg 197341, Russia; radovskiy_am@almazovcentre.ru (A.M.R.); bautin_ae@almazovcentre.ru (A.E.B.); marichevalexandr@gmail.com (A.O.M.); osoff@mail.ru (V.V.O.); natyciel87@gmail.com (N.Y.S.); barbosachka85@gmail.com (L.A.M.)

**Keywords:** nitric oxide, cardiopulmonary bypass, acute kidney injury, liver injury, cardiac surgery, histopathology

## Abstract

**Objective**. To evaluate the effect of NO added to the sweep gas of the oxygenator during cardiopulmonary bypass (CPB) on the liver and kidneys in pigs. **Methods**. An experiment was carried out on 10 pigs undergoing cardiac surgery using CPB. NO was added to the sweep gas of the oxygenator at a concentration of 100 ppm for the animals in the experimental group (CPB-NO, *n* = 5). Animals in the control group (CPB-contr, *n* = 5) did not receive NO in the sweep gas of the oxygenator. The CPB lasted 4 h, followed by postoperative monitoring for 12 h. To assess the injury to the liver and kidneys, the levels of alanine aminotransferase (ALT), aspartate aminotransferase (AST), bilirubin, creatinine, and neutrophil gelatinase-associated lipocalin (NGAL) were determined initially, at weaning from the CPB, and 6 and 12 h after weaning from the CPB. The glomerular filtration rate (GFR) was evaluated initially, at weaning from the CPB, and 6 and 12 h after weaning from the CPB. A pathomorphological study of the liver and kidneys was performed using semiquantitative morphometry. **Results**. The long four-hour period of CPB deliberately used in our experiment caused liver and kidney injury. In the CPB-contr group, an increase in the ALT concentration was found: 43 (34; 44) U/L at baseline to 82 (53; 99) U/L 12 h after CPB, *p* < 0.05. The AST concentration in the CPB-contr group increased from 25 (17; 26) U/L at baseline to 269 (164; 376) U/L 12 h after CPB, *p* < 0.05. We found no significant increase in the ALT and AST concentrations in the CPB-NO group. There were no significant differences in ALT and AST concentrations between the CPB-NO and CPB-contr groups at all the study time-points. In the CPB-contr group, an increase in the creatinine level was found from 131 (129; 133) µmol/L at baseline to 273 (241; 306) µmol/L 12 h after CPB, *p* < 0.05. We found no significant increase in creatinine level in the CPB-NO group. Creatinine levels in the CPB-NO group were significantly lower than in the CPB-contr group 12 h after weaning from CPB: 183 (168; 196) vs. 273 (241; 306) µmol/L; *p* = 0.008. The GFR in the CPB-NO group was significantly higher than in the CPB-contr group 6 h after weaning from CPB: 78.9 (77.8; 82.3) vs. 67.9 (62.3; 69.2) mL/min; *p* = 0.016. GFR was significantly higher in the CPB-NO group than in the CPB-contr group 12 h after weaning from CPB: 67.7 (65.5; 68.0) vs. 50.3 (48.7; 54.9) mL/min; *p* = 0.032. We found no significant differences between the study groups in the level of NGAL. We found several differences between the groups in the pathomorphological study. **Conclusions**. NO added to the sweep gas of the oxygenator reduces creatinine levels and increases GFR during prolonged CPB injury. Further research is required.

## 1. Introduction

Cardiopulmonary bypass (CPB) is required in most cardiac surgery procedures. The incidence of liver injury after cardiac surgery using CPB is approximately 10% of patients [1,2]. Many patients undergoing cardiac surgery already have chronic liver disease [3,4]. Usually, CPB-related liver injury is accompanied by hyperbilirubinemia and an increase in transaminase levels, with further recovery within a few days [5]. Hyperbilirubinemia after cardiac surgery, occurring with a frequency of 6.5–20%, is associated with an increased risk of death [6]. The incidence of hepatic failure after cardiac surgery is as low as 0.1%, but it is associated with a high mortality rate (74%) [7]. Acute kidney injury (AKI) is a common complication of cardiac surgery using CPB. Postoperative AKI develops in 18.2–30% of patients undergoing cardiac surgery. This complication is associated with prolonged hospitalization and increased mortality and risk of stroke [8,9,10,11]. Renal replacement therapy is used in 1–2.9% of patients after cardiac surgery with CPB [8,12]. The 30-day mortality rate in patients undergoing renal replacement therapy after cardiac surgery with CPB has been reported as 42% [8]. Thus, due to CPB-related liver and kidney injury associated with high mortality, new approaches to the prevention and treatment of these complications are required.

One of the negative effects of CPB is hemolysis with the release of toxic oxyhemoglobin into the blood plasma [13]. One of the ways to utilize cell-free hemoglobin is its oxidation with nitric oxide (NO) to form methemoglobin. Thus, increased consumption of NO during CPB leads to depletion of its reserves, vasoconstriction, and an increased systemic inflammatory response [14].

NO is one of the key regulators of physiological systems and metabolic processes, functioning in all tissues and organs. It is secreted in response to shear stress via healthy endothelial cells, where it acts as a major vasodilator. This effect is determined by the NO-mediated activation of soluble guanylate cyclase, which catalyzes the formation of c-GMP and ultimately leads to vasodilation [15]. In addition, NO reduces smooth muscle cell proliferation, platelet aggregation, and endothelial leukocyte binding. Consequently, it has a protective effect on blood vessels and participates in vascular homeostasis [16]. NO added to the sweep gas of the oxygenator during CPB can reduce organ injury in cardiac surgery. The results of several clinical studies have reported on the nephroprotective effects of NO added to the sweep gas of the oxygenator [17,18]. Christopher James et al. reported that NO added to the sweep gas of the oxygenator during CPB in children reduced the risk of low cardiac output syndrome to varying degrees [19]. In our experiment, we studied the hepatoprotective and nephroprotective effects of NO added to the sweep gas of the oxygenator during prolonged CPB.

The objective of the study: to evaluate the effect of NO added to the sweep gas of the oxygenator during CPB on the liver and kidneys in experimental pigs.

## 2. Methods

The study was approved by the Bioethical Committee of the Almazov National Medical Research Center (Protocol No. ПЗ_22_6_V2 dated 6 August 2022). Our study was a pilot, so we did not estimate a target sample size. A total of 10 female domestic pigs (Landrace) aged from 3 to 4.3 months were included in the study. The median body weight was 38.9 (37.7; 40.9) kg. The animals were divided into two groups. For the animals in the experimental group (CPB-NO, *n* = 5), NO was added to the sweep gas of the oxygenator at a concentration of 100 ppm during CPB. The animals in the control group (CPB-contr, *n* = 5) did not receive NO in the sweep gas of the oxygenator. All animals underwent the CPB for 4 h with further postoperative monitoring for 12 h. This high duration of the CPB was chosen to induce more pronounced liver and kidney injury.

### 2.1. Anesthesia and Cardiopulmonary Bypass

General anesthesia was performed, supplemented by intercostal nerve blockade. Premedication included intramuscular administration of zolazepam/tiletamine (Zoletil Virbac, Carros, France) at a dosage of 20 mg/kg. The peripheral vein (ear vein) was punctured and catheterized with an 18 and 20 G catheter. After induction of anesthesia with propofol (Propofol-lipuro, B. Braun, Melzungen, Germany) at a dose of 2–3 mg/kg, direct laryngoscopy and tracheal intubation were performed. After tracheal intubation, the non-depolarizing muscle relaxant rocuronium bromide (Kruaron Veropharm, Moscow, Russia) was administered at a dose of 0.6–1.2 mg/kg. Anesthesia was maintained via inhalation of isoflurane (Aurun Baxter healthcare corporation, Deerfield, IL, USA) using a Heyer Medical AG vaporizer (Drager, Lubeck, Germany) at a dosage of 1.5–2.5 vol.%. All animals underwent jugular vein catheterization. Invasive monitoring of blood pressure (BP) was carried out by catheterization of the femoral artery with a B. Braun 20 G catheter (B. Braun, Germany). In order to control diuresis, the bladder was catheterized with a Nelaton 10 Fr catheter.

Monitoring was carried out using the Mindray BeneView T8 monitor system (Mindray, Shenzhen, China). Monitoring during the experiment included pulse oximetry, electrocardiography (ECG), measurement of central temperature, invasive BP and central venous pressure (CVP), gas analysis, and respiratory rate. Respiratory support was performed in the modes of normoxia and normocapnia. The Mindray Wato Ex-35 (Mindray, Shenzhen, China) was used for ventilation. We used tidal volume = 8 mL/kg, respiratory rate = 10/min, PEEP = 5 cm H_2_O, FiO_2_ = 0.45. Subsequently, respiratory support parameters were adjusted according to blood gases.

Before the surgical procedure, the intercostal nerves were blocked with ropivacaine (Ropivacaine, Pharmaceutical Protection, Khimki, Russia), at a rate of 5 mg/kg.

CPB was performed using a WEL-1000B plus cardiopulmonary bypass machine (Tianjin Welcome Medical Equipment, Tianjin, China) and Inspire Sorin oxygenators (Milan, Italy). CPB-priming included Helofusin 500 mL (B. Braun, Melzungen, Germany), Sterofundin 500 mL (B. Braun, Melzungen, Germany), heparin (Heparin sodium, B. Braun, Melzungen, Germany) at the rate of 3 units/mL of priming, and sodium bicarbonate (Dal’khimfarm, Khabarovsk, Russia) to normalize the pH at the rate of 3 mmol/100 mL of priming. Before the start of CPB, heparin was administered at a dose of 300 units/kg. CPB started at the activated clotting time (ACT) > 480 s. The perfusion index was 2.5 L/min/m^2^. The mean arterial pressure (MAP) was maintained at 60–80 mmHg. The heart was unloaded throughout the CPB. The CVP was 0–2 mmHg. The initial gas flow was 2 L/min, then was corrected based on blood gases. The blood–gas mixture was controlled in the α-stat mode. Normothermia was maintained using a heat exchanger connected to the oxygenator with a target temperature of 37.5–38 °C. During CPB, general intravenous anesthesia with propofol was performed at a dosage of 10–20 mg/kg/hour. Weaning from CPB was performed after reaching an MAP of 90–100 mmHg and a CVP of 8–12. We avoided reversion of heparin through use of protamine sulfate, preferring thorough surgical hemostasis in these procedures.

In the postoperative period, all animals underwent prolonged inhalation anesthesia with isoflurane at a dosage of 1.2–1.5% (1 MAC). Meloxicam (Meloxicam Canonpharma, Shchelkovo, Russia) was used for anesthesia at a dosage of 0.4 mg/kg. Muscle relaxants were not routinely used in the postoperative period. For the analysis of hemodynamics, the Fick cardiac index (CI), blood pressure, CVP, systemic vascular resistance (SVR), and SvO_2_ were evaluated. With reduced preload (CVDP < 8 mmHg), SvO_2_ > 60% in combination with arterial hypotension (MAP < 65 mmHg) and normal SVR, infusion was performed. For infusion therapy, a crystalloid solution of Sterofundin was used, as well as sodium bicarbonate in the case of metabolic acidosis. Inotropic support was initiated in the case of arterial hypotension (MAP < 65 mmHg) in combination with reduced CI (CI < 2.5 L/min/m^2^) and SvO_2_ < 60%. Dopamine (Ellara, Voscow, Russia) was a first-line inotropic agent (dosage 4–8 mcg/kg/min). In case of dopamine’s ineffectiveness, epinephrine (Ellara, Voscow, Russia) was used (dosage 0.03–0.1 mcg/kg/min). In the presence of arterial hypotension caused by systemic vasoplegia, vasopressor support with norepinephrine (Ellara, Voscow, Russia) was initiated at a dosage necessary to maintain MAP > 65 mmHg. Protective ventilation was performed.

### 2.2. Surgical Procedure

A left-sided thoracotomy was performed on 3 intercostals. An aortic cannula 20 Fr (Medtronic, Dublin, Ireland) was inserted into the ascending aorta and a venous cannula 31 Fr (Medtronic, Dublin, Ireland) was inserted into the right atrium. After weaning from the CPB and removing the cannula, hemostasis was performed. Drainage was installed into the left pleural cavity and the pericardial cavity. Layered suturing of the wound was performed.

### 2.3. Adding NO to the Sweep Gas of the Oxygenator

**Figure 1:**
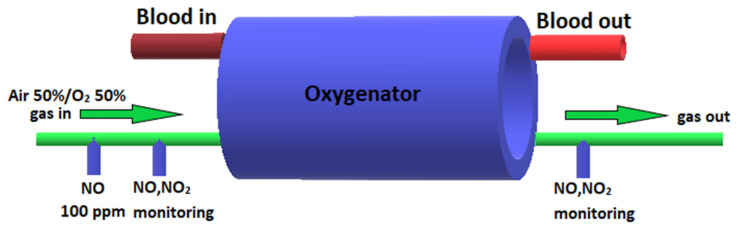
Scheme of supply of air–oxygen mixture, NO, and monitoring of NO and NO_2_ to the oxygenator.

In the CPB-NO group of animals, NO synthesized in the experimental device was added to the sweep gas of the oxygenator at a dosage of 100 ppm during CPB. Two stopcock systems (Discofix C, B. Braun, Melzungen, Germany) were installed in the inlet trunk: the NO supply line was connected in the first stopcock system (at a distance of 10 cm to the oxygenator); and a line for monitoring NO and NO_2_ was connected to the second stopcock system (at a distance of 5 cm to the oxygenator). In addition, NO and NO_2_ were monitored 5 cm after the oxygenator (Figure 1). Animals from the CPB-contr group did not receive NO. The upper limit of the safe NO_2_ concentration was determined as 2 ppm. When NO_2_ exceeded more than 2 ppm, the NO supply was automatically stopped.

### 2.4. Assessment of Liver and Renal Function

To assess liver and renal function, the level of alanine aminotransferase (ALT), aspartate aminotransferase (AST), bilirubin, creatinine, and neutrophil gelatinase-associated lipocalin (NGAL) levels were determined initially, immediately after weaning from the CPB and 6 and 12 h after weaning from the CPB. To evaluate the glomerular filtration rate (GFR), we used this equation: GFR = 1.879 × BW^1.092^/P cr ^0.6^ [20].

### 2.5. Pathomorphology

All animals were subjected to an autopsy at an interval of 15–30 min after euthanasia. After daily fixation of the selected tissue in a 10% solution of neutral formalin, slices were excised for histological examination. Two pieces from the left lobe of the liver were excised from the liver tissue, having a color of different intensity. Two pieces were also excised from the kidney tissue (closer to the surface and the pelvis).

Dehydration and paraffin embedding were carried out using an Excelsior AS automatic histological processor (Thermo Fisher Scientific, Waltham, MA, USA) in a ready-made IsoPREP solution (Biovitrum, Saint-Petersburg, Russia) and a HISTOMIX paraffin medium (Biovitrum, Saint-Petersburg, Russia). Using a rotary microtome NM 325 (Thermo Fisher Scientific, Waltham, MA, USA), sections with a thickness of 2–3 microns were created, which were further dewaxed, dehydrated, and stained with hematoxylin-eosin (H-E). Microscopic examination was carried out using a Nikon NiE microscope (Tokyo, Japan) at magnifications of ×40, ×100, ×200, and ×400.

To analyze the histological changes, dystrophic changes in the parenchyma and stroma cells, circulatory disorders, and inflammation were evaluated. The assessment was carried out semi-quantitatively in scores from 0 to 3, where scores indicated 0—lack of severity of the trait, 1—mild severity, 2—moderate severity, and 3—strong severity. The results of the semi-quantitative histological study were compared with the data from biochemical tests in the animals. To assess the number of binucleated hepatocytes, liver samples from an animal that had not undergone either cardiac surgery or CPB (Control-noCPB) were examined.

### 2.6. Statistical Analysis

Statistical analysis was performed using the MedCalc Statistical Software package 20.218 (MedCalc Software Ltd., Ostend, Belgium). Due to the small sample size, nonparametric methods were used. To compare quantitative variables, the Mann–Whitney U-test for independent groups and the Wilcoxon criterion for dependent groups were used. A multi-group comparison of quantitative variables was performed using the Kruskal–Wallis criterion. Comparisons of qualitative variables were carried out using the exact Fisher criterion. The data are presented as a median (Q1; Q3). The critical level of significance was considered as *p* = 0.05.

## 3. Results

We did not find statistically significant differences in the main parameters of hemodynamics, gas exchange, and perfusion in the studied groups (Table 1). During the experiment, all animals had a predominant sinus rhythm. Hemodynamically, no significant atrial and ventricular extrasystoles were displayed at the surgical stage. There were no ischemic changes on the ECG.

Data on the vasoactive inotropic index (VIS) are presented in Table 2. We found no differences between the study groups in the doses of inotropic and vasoactive drugs during the experiment.

Hematological parameters: no differences were found between the studied groups in hematological parameters during the experiment (Table 3).

A typical significant increase in methemoglobin levels was found with the nitric oxide (Table 4). Methemoglobin was significantly higher in the CPB-NO group compared to the CPB-contr group at the end of cardiopulmonary bypass and 6 h after cardiopulmonary bypass.

### 3.1. Assessment of Liver Function

**Figure 2:**
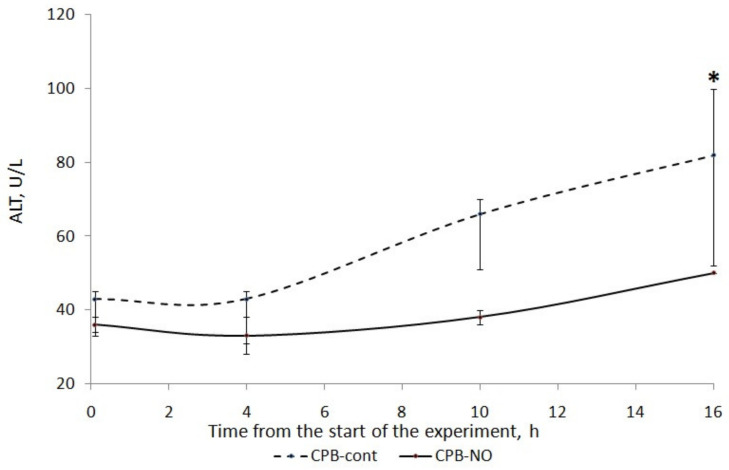
ALT dynamics in animals of the studied groups. The data are presented as a median (Q1; Q3); *—*p* < 0.05 in comparison with the initial values.

**Figure 3:**
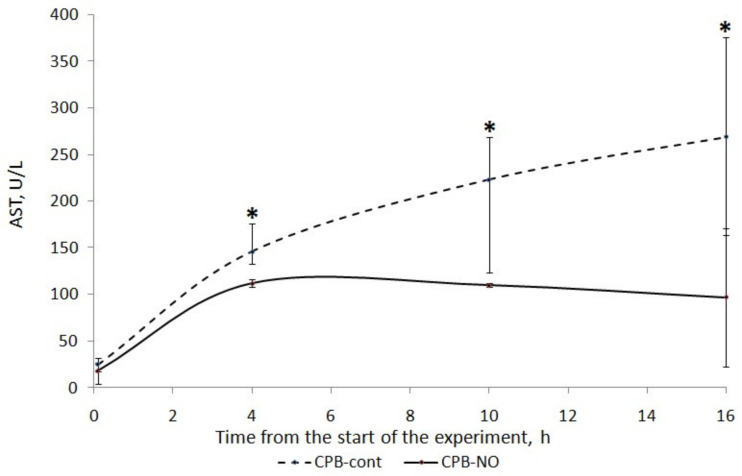
Dynamics of AST in animals of the studied groups. The data are presented as a median (Q1; Q3); *—*p* < 0.05 in comparison with the initial values.

A long four-hour period of CPB, deliberately used in our experiment, caused injury to the liver of the animals. There was an increase in ALT and AST in the postoperative period (Figure 2 and Figure 3).

We found a less pronounced increase in ALT and AST in animals that received NO during CPB. In the CPB-contr group, the ALT level significantly exceeded the baseline 12 h after the weaning from the CPB. There was no significant increase in ALT in the CPB-NO group. In animals of the CPB-contr group, the AST levels after weaning from the CPB were significantly higher than the baseline values. When using NO, there were no differences in the AST in comparison with the initial values.

When comparing the ALT level between the CPB-contr and CPB-NO groups, a tendency to exceed this indicator was revealed in the control group (Table 5). However, these differences were not significant due to the small number of samples. In the CPB-contr group, a tendency toward higher values of the area under the curve (AUC) of the dynamics of ALT concentration and the maximum ALT value for each animal were noted.

We found a significant increase in the AST level compared to the baseline level in the CPB-contr group at the weaning from the CPB, and after 6 and 12 h (Table 6). In addition, trends towards higher values of the AUC AST and the maximum AST level for each animal were found. However, these differences were not statistically significant.

Table 7 presents data on the level of bilirubin in the experimental animals. We found no significant differences between the study groups.

### 3.2. Pathology of the Liver

**Figure 4:**
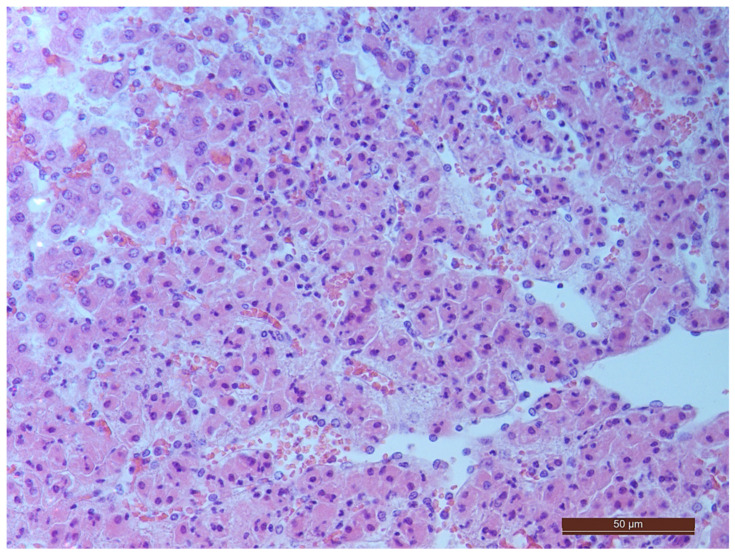
Aggregation of erythrocytes in an animal from the CPB-contr group. Staining with H-E, 400×.

**Figure 5:**
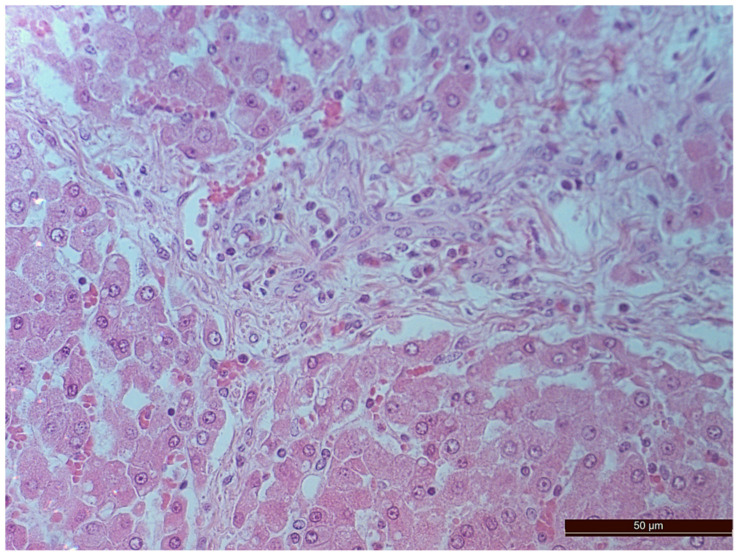
Cells with bright-eosinophilic cytoplasm in the portal tract of an animal from the CPB-contr group. Staining with H-E, 400×.

**Figure 6:**
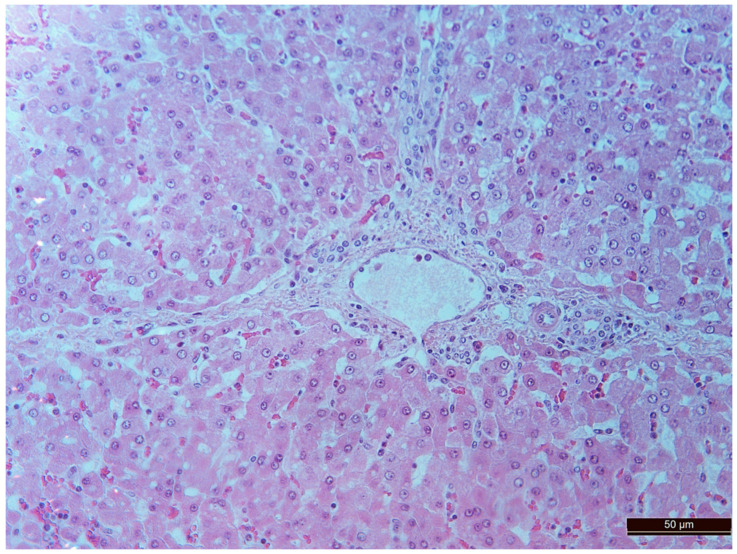
A vessel in the area of the expanded portal tract with moderate mononuclear infiltration in an animal of the CPB-contr group. Staining with H-E, 400×.

**Figure 7:**
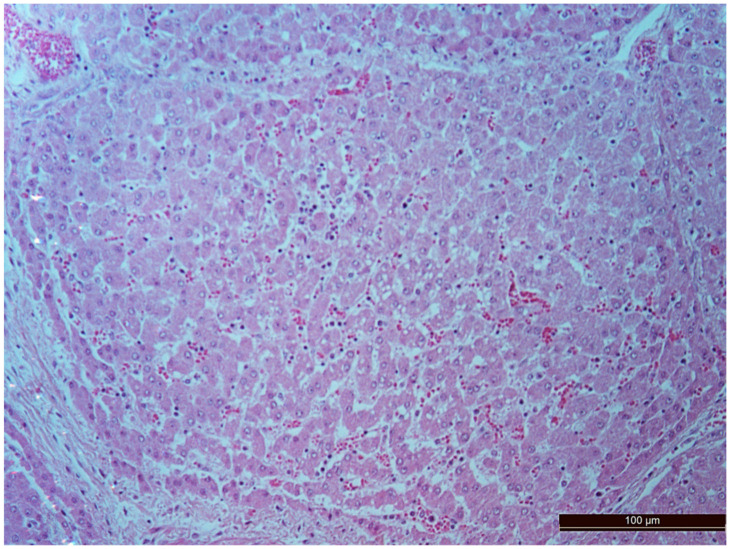
Diffuse infiltration by neutrophils and lymphocytes in an animal of the CPB-contr group. Staining with H-E, 200×.

A total of 55 histological preparations obtained from five animals of the CPB-contr group, from five animals of the PB-NO group and two from animals not subjected to either cardiac surgery or CPB (Control-noCPB) were studied. The pathological examination of the livers of animals from the control group revealed capsule thickening, edema, and inflammatory infiltration by mononuclear cells of moderate severity. The hepatic beams were thickened and some hepatocytes had nuclei shifted to the periphery. The binucleated cells were counted in both the central part and on the periphery of the lobule (Table 8). Their number was 1–4 cells in the field of view. The cytoplasm of hepatocytes has a different color intensity, which demonstrates its pronounced vacuolization. Extensive areas of diffuse infiltration by lymphocytes and neutrophils are observed, localized around the vessels and sinuses. The vessel walls are thickened, edematous, and with moderate lymphocytic infiltration. Endothelial cells are locally disintegrated. There is a marked fullness with aggregation of erythrocytes up to the formation of “coin columns”. In addition, focal expansion of sinusoids was noted. The perisinusoidal cells have enlarged nuclei and bulge out into the lumen. These changes are considered to be morphological signs of liver dysfunction and are shown in Figure 4, Figure 5, Figure 6 and Figure 7.

**Figure 8:**
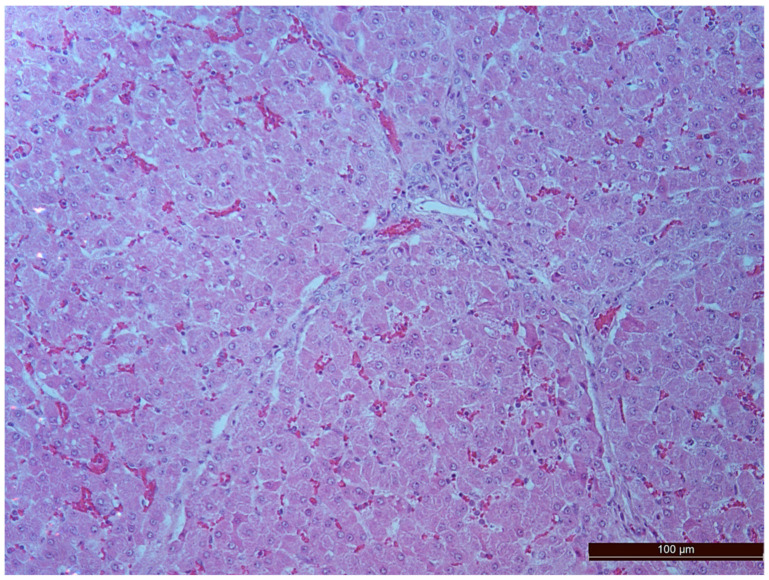
Binuclear hepatocytes in an animal of the CPB-NO group. Staining with H-E, 200×.

In animals from the CPB-NO group, the degree of liver injury was significantly less in all the compared parameters. In all liver samples, there was an increase in the number of binucleated cells, which can be considered to be an indirect sign of increased functional activity (Figure 8, Table 4). However, the differences between the groups were not statistically significant, probably due to the small number of samples.

### 3.3. Renal Function

**Figure 9:**
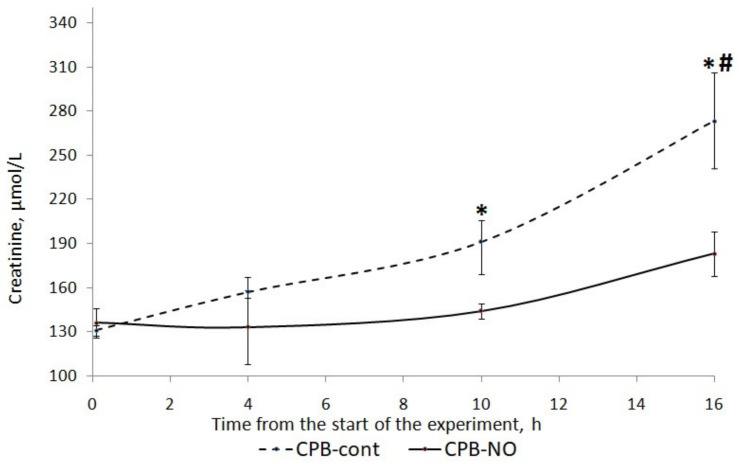
Dynamics of creatinine in animals of the studied groups. The data are presented as a median (Q1; Q3). *—*p* < 0.05 in comparison with the initial values; #—*p* < 0.01 in an intergroup comparison.

After weaning from the CPB, an increase in creatinine levels was found in both groups. In the CPB-contr group, the increase in creatinine levels compared with baseline values was statistically significant (Figure 9). The increase in creatinine levels in the CPB-NO group was not statistically significant.

When comparing creatinine levels between the CPB-contr and CPB-NO groups (Table 9), a tendency to its higher values was found in animals of the control group (Table 8). In addition, 12 h after weaning from the CPB, these differences were statistically significant (*p* = 0.008).

When comparing the glomerular filtration rate (GFR) between the CPB-contr and CPB-NO groups (Table 10), significantly higher levels of GFR were found in animals of the CPB-NO group at 6 and 12 h after weaning from the CPB. Significantly higher urine output during surgery was found in the CPB-NO group.

Hemolysis in the study groups was negligible (Table 11). There were no significant differences in the content of free plasma hemoglobin between the studied groups.

**Figure 10:**
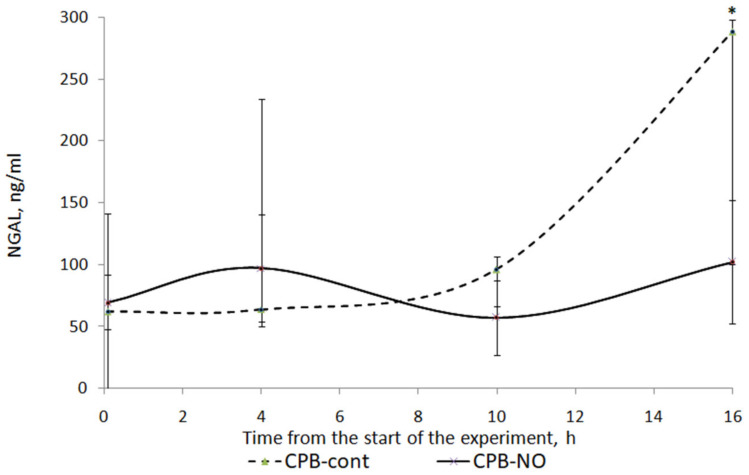
Dynamics of NGAL in animals of the studied groups. The data are presented as a median (Q1; Q3). *—*p* < 0.05 in comparison with the initial values.

Figure 10 shows a graph of NGAL level dynamics in animals from the studied groups. When NO was added to the sweep gas of the oxygenator, there was a weakly pronounced tendency to increase the level of NGAL in the postoperative period. In animals of the CPB-contr group, a tendency was found to increase the level of NGAL in comparison with the initial values. At the time point 12 h after weaning from the CPB, these differences were statistically significant.

When comparing the NGAL level between the CPB-contr and CPB-NO groups, a tendency to higher values of the indicator was found in animals of the control group. However, these differences were not statistically significant due to the small sample size (Table 12).

### 3.4. Pathology of Kidneys

**Figure 11:**
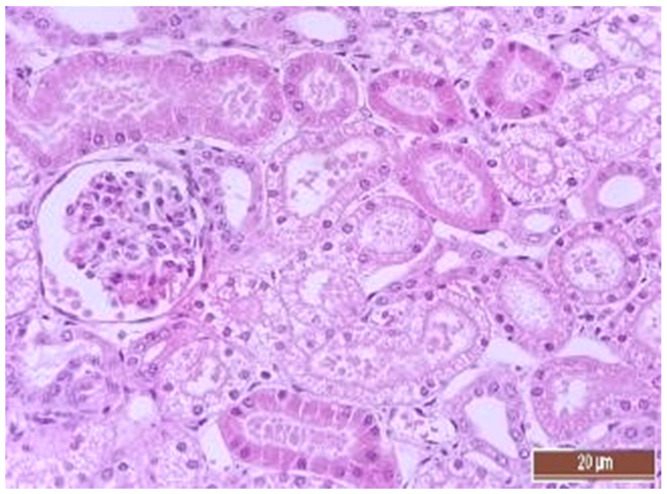
Alterative changes in the tubule epithelium in the renal cortex of a pig from the CPB-contr group (2 points, moderate changes). Staining with H-E, 400×.

**Figure 12:**
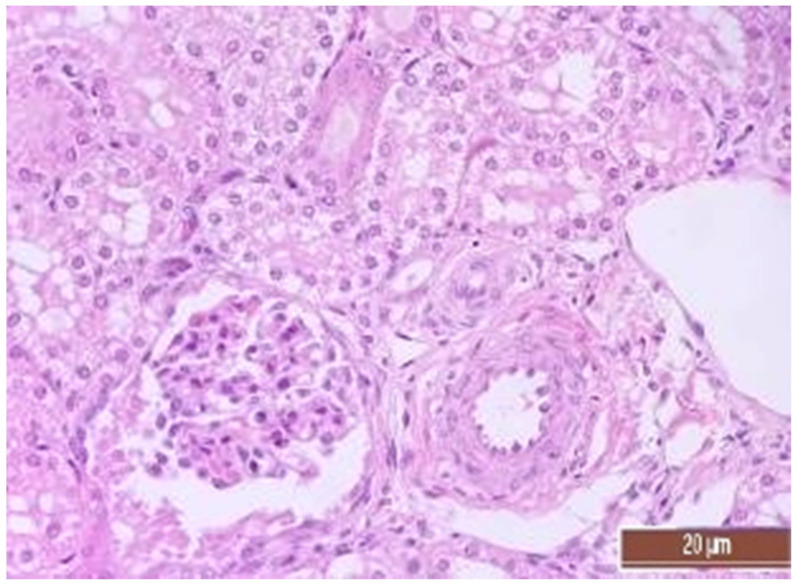
Alterative changes in the tubule epithelium in the renal cortex of the pig from the CPB-NO group (1 point, mild changes). Staining with H-E, 400×.

**Figure 13:**
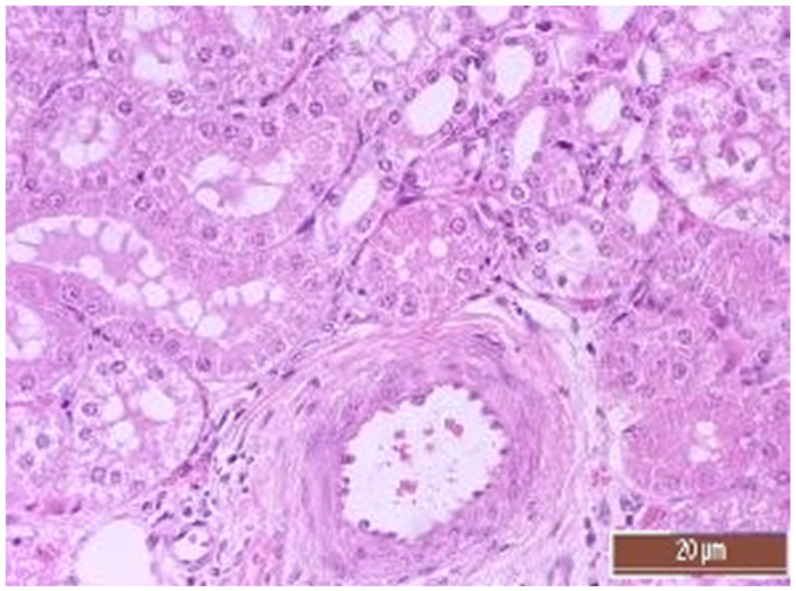
Alterative changes in the vessel endothelium (2 points, moderate changes). Staining with H-E, 400×.

**Figure 14:**
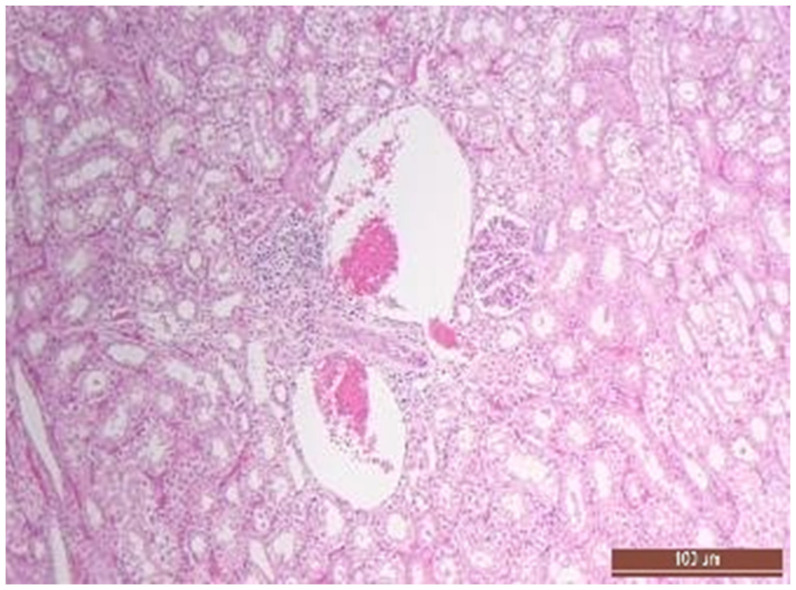
Focal predominantly lymphocytic infiltration of the renal cortex in an animal of the CPB-contr group. Staining with H-E, 100×.

**Figure 15:**
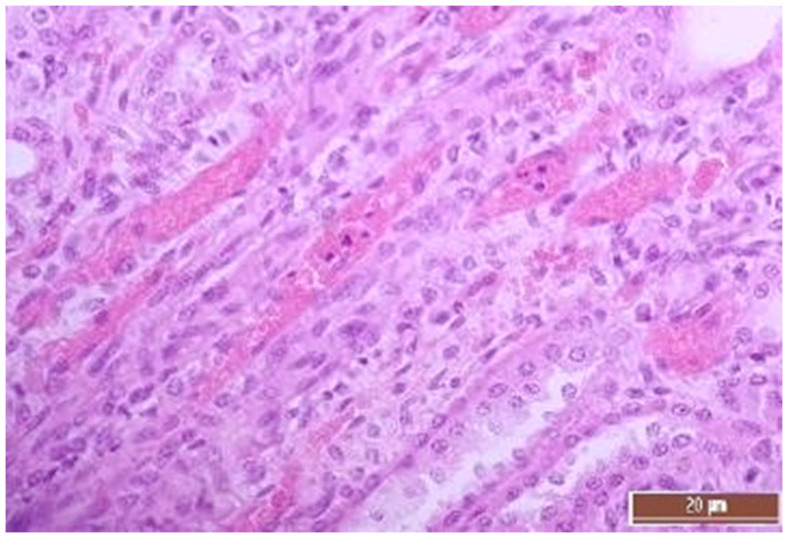
Hyperemia of capillaries in renal medulla in an animal of the group, CPB-contr, erythrocytes forming “coin columns” (3 points, severe changes). Staining with H-E, 400×.

**Figure 16:**
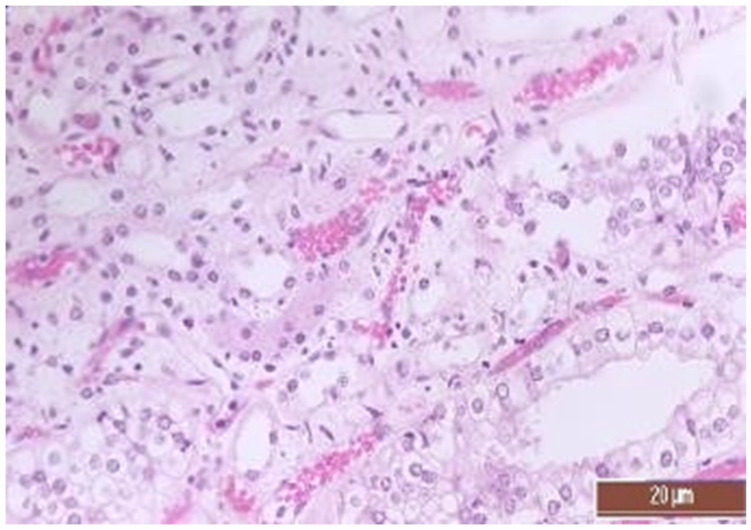
Hyperemia of capillaries in renal medulla in an animal of the CPB-NO group (2 points, moderate changes) Staining with H-E, 400×.

A total of 50 histological preparations obtained from five animals of the CPB-contr group and from five animals of the CPB-NO group were studied. During the pathological examination of the kidneys of animals of both groups, the cortices and medullae of the kidneys were differentiated, without macroscopic signs of structural disorders, and the pelvises were not expanded. The capsules were not thickened. Microscopic examination of animals from the control group showed moderate and pronounced vascular disorders and injuries (Table 13, Figure 11, Figure 12, Figure 13, Figure 14, Figure 15 and Figure 16). Degenerative epithelial changes of two types were observed in the tubules of the cortical substance, partly with vacuolization and enlightened cytoplasm, partly with bright eosinophilic cytoplasm locally in the lumens. Locally, there were non-nuclear epithelial cells of tubules, mainly convoluted; additionally, tubules were collected with weakly expressed damage to epithelial cells. Expansion and fullness of the vascular lumens, which were more pronounced in the capillaries of the tubulointerstitia and medullae, were found. Erythrocyte sludge was noted, in the form of “coin columns”. The fullness of the capillaries of the glomeruli was unexpressed. The walls of the vessels were thickened, the endothelium of the arteries and arterioles showed dystrophic changes, they were swollen, and the nuclei were rounded. There were also isolated local lymphoplasmocytic infiltrates in the cortical substance, mainly near the vessels. Moderate and severe ischemic lesions and necrosis sites were not detected. These morphological changes correspond to acute, mild kidney injury.

The same histological changes were found in animals from the CPB-NO group, but with a lower degree of severity of damage (Table 7). Thus, alterative changes in the epithelium of the tubules were scored as three points in only one animal. In the CPB-contr group, changes of three points were detected in two animals. According to the results of the quantitative assessment, the median of alterative changes in the tubule epithelia in the control group was two points. In the CPB-NO group the median of alternative changes was one point. No statistical significance was revealed due to the small number of samples. Vascular disorders comparable to the control group were also found in the CPB-NO group. The expansion and fullness of the vessels of the cortices and medullae were denoted by two and three points (moderate and severe). In the lumens of the capillaries, erythrocyte sludge was observed in the form of “coin columns” and alterative changes in the vascular endothelia were noted by an average of two points (moderate and severe). In the CPB-NO group, as well as in the control group, single lymph-plasmocytic infiltrates in the cortical substance were observed. Moderate and severe ischemic lesions and necrosis sites were not detected. Morphological changes in the kidney tissue of both groups are shown in Figure 11, Figure 12, Figure 13, Figure 14, Figure 15 and Figure 16.

## 4. Discussion

For the first time, we have studied the hepatoprotective effects of NO added to the sweep gas of the oxygenator during surgery. The obtained results demonstrated liver injury as a result of long duration CPB. In the animals of the control group, a statistically significant increase in markers of liver injury was found in comparison with the preoperative level. In cases of NO supply to the sweep gas of the CPB oxygenator, there was only a weakly pronounced tendency to increase markers of injury, without statistical significance. A comparison of the CPB-NO and CPB-contr groups demonstrated a tendency to a higher level of damage markers in the animals of the control group; however, the results were not statistically significant, which was probably due to the limited sample size.

The results of the assessment of the effect of nitric oxide on kidney function demonstrated a positive effect of NO added to the sweep gas of the oxygenator during surgery. Creatinine levels were significantly lower in the CPB-NO group 12 h after weaning from CPB. Our data confirm the results obtained in previous studies on the effect of NO added to the sweep gas of the oxygenator on renal function. For example, in two studies, the use of NO added to the sweep gas of the oxygenator at dosages of 40 and 80 ppm was associated with a reduction in the risk of AKI during cardiac surgery in adults [17,18]. At the same time, a large multicenter trial in pediatric surgery did not report the protective effects of nitric oxide on renal function. However, as the authors note, this could be due to the low dosage of nitric oxide (20 ppm), as well as the fact that some patients from the control group received inhaled NO [21].

In accordance with modern ideas, organ dysfunction during surgical procedures using CPB is the result of several interrelated factors. Among these factors are the syndrome of systemic inflammatory responses, ischemic and reperfusion injury, as well as damage to the intestinal–mucosal barrier with the occurrence of bacterial translocation and endotoxemia [5,22,23]. Such changes lead to impaired microcirculation and damage to the endothelium—one of the key components of the pathophysiology of ischemic and reperfusion injury. Increased permeability of endothelial cells promotes the migration of activated leukocytes into tissues, with additional damage to blood vessels and parenchyma of organs [24,25,26].

The results of numerous studies confirm the protective effects of NO in conditions of ischemia-reperfusion and systemic inflammatory reaction. NO has antiplatelet and antineutrophilic effects, which reduce the inflammatory response to ischemic and reperfusion injury and protect the endothelium [27]. In addition, NO has anti-apoptotic effects due to the inactivation of the enzyme caspase-3. Another possible protective mechanism is the NO-dependent synthesis of cGMP, which can protect against reperfusion injury by increasing blood flow. It is also assumed that NO-mediated activation of protein kinase G along the sGC/cGMP pathway opens mitochondrial K-ATP channels, thereby reducing the accumulation of calcium in the mitochondria and preventing the loss of cytochrome C from the intermembrane space of mitochondria [28]. The organ protective effects of NO during reperfusion have been demonstrated in experimental studies. For example, it was shown that inhalation of NO for 24 h in mice after ligation of the left anterior descending coronary artery with subsequent reperfusion, was accompanied by a decrease in the myocardial infarction zone and an improvement in left ventricular function [29].

There is evidence that NO deficiency occurs during ischemia-reperfusion [30]. It is assumed that early endothelial damage worsens the production of NO, which eliminates the endogenous antineutrophilic effects of NO [31,32]. Along with a decrease in NO production, there is an increase in its consumption due to the reaction with reactive oxygen species, with the further formation of toxic peroxynitrite.

Surgical procedures using CPB are accompanied by hemolysis. As a result of increased consumption of NO, endothelial dysfunction develops, which leads to an increase in systemic vascular and pulmonary resistance with impairment of the microcirculation [33]. There is evidence that hemolysis associated with CPB can lead to AKI and intestinal tissue damage [34,35]. In addition, cell-free hemoglobin may become the biomarker of AKI [36]. Thus, one of the main, supposed protective mechanisms of NO when added to the sweep gas of the oxygenator is the scavenging of cell-free hemoglobin, as well as the reduction in levels of nitric oxide deficiency. In the study of S. Minni et al., the authors reported a reduction the negative effects of intravascular hemolysis via inhalation of 80 ppm of NO. The inhaled NO scavenged up to 85–90% of the total amount of hemoglobin to methemoglobin, thereby inhibiting endogenous NO scavenging via cell-free hemoglobin [37].

The method of adding NO to the sweep gas of the CPB oxygenator for organ protection requires further study. The results of our study are preliminary and not conclusive because of the limited sample size of the animals that may underpower the study’s findings. Therefore, the results should be interpreted as hypothesis generating. In our opinion, future research should focus on studying the safety of the technique, in particular, assessing nitrosative stress. In addition, it is necessary to select the optimal dosage of NO, and to identify indications and contraindications to the use of this technique.

## 5. Study Limitations

The main limitation of our study was the small sample size. Our study was a pilot, so we did not previously estimate the sample size. Some of the differences between groups were not identified, probably because the sample size was insufficient.

The serious limitation of the study is that it was performed on healthy animals that did not have impaired visceral organ perfusion. In patients with vascular disease and impaired visceral perfusion, the organoprotective effects of nitric oxide may be less pronounced.

No part of the study has been previously published.

## 6. Conclusions

NO added to the sweep gas of the oxygenator during surgery reduces creatinine levels and increases the glomerular filtration rate during prolonged CPB injury. Further research is required, including on the methodology and safety of this technique.

## Figures and Tables

**Table 1:** The main parameters of hemodynamics, gas exchange and perfusion in the studied groups, median (Q1; Q3), *n* = 10.

Parameters	Point	CPB-contr, *n* = 5	CPB-NO, *n* = 5	Mann–Whitney U-Test
MAP, mm Hg	Baseline	75 (67.5; 85)	78 (75; 81)	*p* = 0.832
	2 h CPB	68 (65; 70)	69 (66; 70)	*p* = 0.833
	Weaning CPB	72 (70; 75)	78 (76.5; 79)	*p* = 0.036
	6 h after CPB	71 (70; 75)	78 (70; 83)	*p* = 0.599
	12 h after CPB	70.5 (70; 79)	75 (72.5; 77)	*p* = 0.834
CVP, mm Hg	Baseline	12 (9.5; 14)	12.2 (8.1; 13)	*p* = 1.000
	2 h CPB	12 (8; 12)	10 (8; 14)	*p* = 0.833
	Weaning CPB	16 (12; 17)	13.6 (12; 15)	*p* = 0.675
	6 h after CPB	13.5 (11; 14.9)	12.2 (12.2; 13.6)	*p* = 0.596
	12 h after CPB	13.8 (13.6; 14.5)	14.6 (13.8; 17)	*p* = 0.195
HR, min^−1^	Baseline	105 (85; 105)	104 (85; 105)	*p* = 0.592
	2 h CPB	90 (85; 95)	92 (85; 95)	*p* = 1.000
	Weaning CPB	103 (100; 105)	96 (93; 104)	*p* = 0.530
	6 h after CPB	92 (75; 95)	98 (95; 106)	*p* = 0.143
	12 h after CPB	97 (95; 108)	94 (90; 107)	*p* = 0.346
FiO_2_	Baseline	0.5 (0.45; 0.5)	0.45 (0.4; 0.5)	*p* = 0.496
	2 h CPB	0.4 (0.4; 0.5)	0.4 (0.45; 0.4)	*p* = 0.513
	Weaning CPB	0.5 (0.4; 0.5)	0.5 (0.5; 0.5)	*p* = 0.513
	6 h after CPB	0.45 (0.45; 0.45)	0.4 (0.4; 0.4)	*p* = 0.174
	12 h after CPB	0.45 (0.45; 0.5)	0.45 (0.45; 0.45)	*p* = 0.343
PaO_2_, mm Hg	Baseline	190 (150; 190)	190 (150; 195)	*p* = 0.827
	2 h CPB	185 (180; 190)	180 (150; 190)	*p* = 0.527
	Weaning CPB	190 (172; 190)	184 (150; 184)	*p* = 0.916
	6 h after CPB	197 (189; 200)	189 (180; 190)	*p* = 0.31
	12 h after CPB	166 (111; 190)	150 (150; 187)	*p* = 1.000
PaCO_2_, mm Hg	Baseline	39 (37; 45.7)	39.1 (36.6; 42.5)	*p* = 0.675
	2 h CPB	34 (33; 34)	35 (33; 35)	*p* = 0.455
	Weaning CPB	38.3 (38; 42.3)	40.7 (33.3; 42.9)	*p* = 0.841
	6 h after CPB	38 (34.9; 41)	38.9 (38; 39.5)	*p* = 0.53
	12 h after CPB	38.3 (35.2; 42)	38 (37; 38.4)	*p* = 0.841
T, °C	Baseline	37 (36.6; 37.3)	36.8 (36.8; 37.4)	*p* = 0.906
	2 h CPB	36 (35.9; 36.2)	36 (35.8; 36.2)	*p* = 1.000
	Weaning CPB	38.5 (38.4; 38.8)	38.6 (38.6; 38.8)	*p* = 0.674
	6 h after CPB	39.4 (39.2; 39.9)	39.6 (39.6; 40.1)	*p* = 0.461
	12 h after CPB	39.5 (39.5; 39.6)	39.4 (39.3; 39.7)	*p* = 0.381
Lactate, mmol/L	Baseline	1.98 (1.27; 2.35)	2.28 (1.81; 3.89)	*p* = 0.548
	2 h CPB	1.55 (1.26; 2.99)	2 (1.75; 4.05)	*p* = 0.31
	Weaning CPB	2.96 (1.83; 3.42)	2.66 (2.06; 3.24)	*p* = 1.000
	6 h after CPB	0.96 (0.72; 1.02)	0.72 (0.39; 1.0)	*p* = 0.566
	12 h after CPB	1.65 (0.82; 2.4)	0.71 (0.66; 0.86)	*p* = 0.064
Drainage blood loss, mL		280 (160; 400)	200 (150; 300)	*p* = 0.69

MAP—mean arterial pressure; CPB—cardiopulmonary bypass; CVP—central venous pressure; HR—heart rate.

**Table 2:** Dynamics of the VIS, in the studied groups, median (Q1; Q3), *n* = 10.

Point	CPB-contr, *n* = 5	CPB-NO, *n* = 5	Mann–Whitney U-Test
Weaning CPB	3 (0; 6)	5 (1.5; 6)	*p* = 1.000
6 h after CPB	4 (0; 7)	3.5 (0.5; 6.5)	*p* = 1.000
12 h after CPB	5 (1.5; 6.5)	3.5 (0.5; 7.5)	*p* = 1.000

CPB—cardiopulmonary bypass.

**Table 3:** The hematological parameters in the studied groups, median (Q1; Q3), *n* = 10.

Parameters	Point	CPB-contr, *n* = 5	CPB-NO, *n* = 5	Mann–Whitney U-Test
Hemoglobin, g/L	Baseline	86 (86; 90)	89 (86; 101)	*p* = 0.597
	2 h CPB	64 (57; 65)	61 (61; 69)	*p* = 0.753
	Weaning CPB	59 (55; 62)	59 (54; 64)	*p* = 0.916
	12 h after CPB	66 (63; 76)	66 (54; 78)	*p* = 0.525
Leukocytes, ×10^9^/L	Baseline	15.4 (15.3; 16.5)	19.5 (19.2; 20.8)	*p* = 0.31
	2 h CPB	10.2 (10.1; 10.6)	11.7 (10.1; 12.8)	*p* = 0.675
	Weaning CPB	12.5 (12.3; 15)	14.9 (10.4; 18.1)	*p* = 1.000
	12 h after CPB	14.2 (12.7; 17.5)	14.9 (11.8; 17.7)	*p* = 0.841
Platelets, ×10^12^/L	Baseline	487 (472; 569)	487 (332; 572)	*p* = 0.834
	2 h CPB	325 (311; 343)	322 (245; 350)	*p* = 0.691
	Weaning CPB	295 (202; 312)	225 (194; 276)	*p* = 0.421
	12 h after CPB	280 (182; 295)	167 (132; 272)	*p* = 0.31

CPB—cardiopulmonary bypass.

**Table 4:** Methemoglobin level in the studied groups (%), median (Q1; Q3), *n* = 10.

Point	CPB-contr, *n* = 5	CPB-NO, *n* = 5	Mann–Whitney U-Test
Baseline (1)	1.95 (1.9; 2)	2.05 (1.95; 2.05)	*p* = 0.4
Weaning CPB (2)	2.2 (1.9; 2.7)	3.2 (3.05; 3.65)	*p* = 0.032
6 h after CPB (3)	1.6 (1.6; 1.8)	2.8 (2.5; 3)	*p* = 0.016
12 h after CPB (4)	1.6 (1.2; 1.9)	2 (1.9; 2.1)	*p* = 0.143
Wilcoxon test	p_1–2_ = 0.715 p_1–3_ = 0.5 p_1–4_ = 0.138	p_1–2_ = 0.043 p_1–3_ = 0.042 p_1–4_ = 0.687	

CPB—cardiopulmonary bypass.

**Table 5:** ALT levels in the studied groups (U/L), median (Q1; Q3), *n* = 10.

Point	CPB-contr, *n* = 5	CPB-NO, *n* = 5	Mann–Whitney U-Test
Baseline (1)	43 (34; 44)	36 (36; 37)	*p* = 0.691
Weaning CPB (2)	43 (31; 45)	33 (29; 38)	*p* = 0.222
6 h after CPB (3)	66 (41; 70)	38 (37; 38)	*p* = 0.222
12 h after CPB (4)	82 (53; 99)	50 (49; 50)	*p* = 0.151
AUC ALT (U/L/16 h)	775 (464; 855)	451 (440; 489)	*p* = 0.175
Wilcoxon test	p_1–2_ = 0.682 p_1–3_ = 0.08 p_1–4_ = 0.043	p_1–2_ = 0.225 p_1–3_ = 1.000 p_1–4_ = 0.08	
Max. ALT (U/L)	82 (53; 99)	50 (49; 50)	*p* = 0.151

Max. ALT—the maximum ALT value noted in each of the animals; CPB—cardiopulmonary bypass; AUC—the area under the curve of the dynamics of ALT concentration.

**Table 6:** AST levels in the studied groups (U/L), median (Q1; Q3), *n* = 10.

Point	CPB-contr, *n* = 5	CPB-NO, *n* = 5	Mann–Whitney U-Test
Baseline (1)	25 (17; 26)	18 (16; 32)	*p* = 0.91
Weaning CPB (2)	146 (129; 174)	112 (99; 116)	*p* = 0.421
6 h after CPB (3)	223 (124; 269)	110 (99; 112)	*p* = 0.175
12 h after CPB (4)	269 (164; 376)	97 (94; 171)	*p* = 0.175
Wilcoxon test	p_1–2_ = 0.043 p_1–3_ = 0.043 p_1–4_ = 0.043	p_1–2_ = 0.043 p_1–3_ = 0.068 p_1–4_ = 0.08	
AUC AST (U/L/16 h)	2325 (1255; 2669)	1094 (1013; 1248)	*p* = 0.117
Max. AST (U/L)	269 (174; 376)	112 (111; 283)	*p* = 0.076

Max. AST—the maximum AST value noted in each of the animals; CPB—cardiopulmonary bypass; AUC—the area under the curve of the dynamics of AST concentration.

**Table 7:** Bilirubin levels in the studied groups (µmol/L), median (Q1; Q3), *n* = 10.

Point	CPB-contr, *n* = 5	CPB-NO, *n* = 5	Mann–Whitney U-Test
Baseline	1.75 (1.59; 2.60)	1.84 (1.51; 8.42)	*p* = 0.752
Weaning CPB	2.60 (2.55; 5.6)	3.65 (3.54; 5.53)	*p* = 0.751
6 h after CPB	2.34 (2.17; 2.87)	2.42 (2.10; 2.52)	*p* = 0.753
12 h after CPB	2.93 (2.39; 3.23)	2.14 (1.70; 2.58)	*p* = 0.352

CPB—cardiopulmonary bypass.

**Table 8:** Assessment of the number of binucleated hepatocytes per 100 cells in groups, median (Q1; Q3), 55 histological preparations from 11 animals.

Localization	Groups	Number of Binuclear Hepatocytes per 100 Cells	Kruskal–Wallis Test
The center of the lobule	CPB-contr, *n* = 25	4 (4; 4)	*p* = 0.170
	CPB-NO, *n* = 25	6 (3; 6)	
	Control-noCPB, *n* = 5	4 (4; 5)	
The periphery of the lobule	CPB-contr, *n* = 25	2 (1; 3)	*p* = 0.426
	CPB-NO, *n* = 25	4 (3; 6)	
	Control-noCPB, *n* = 5	3 (3; 5)	

**Table 9:** Creatinine levels in the studied groups (µmol/L), median (Q1; Q3), *n* = 10.

Point	CPB-contr, *n* = 5	CPB-NO, *n* = 5	Mann–Whitney U-Test
Baseline (1)	131 (129; 133)	136 (129; 145)	*p* = 0.42
Weaning CPB (2)	157 (154; 167)	133 (130; 157)	*p* = 0.291
6 h after CPB (3)	191 (169; 215)	146 (141; 148)	*p* = 0.076
12 h after CPB (4)	273 (241; 306)	183 (168; 196)	*p* = 0.008
Wilcoxon test	p_1–2_ = 0.08 p_1–3_ = 0.043 p_1–4_ = 0.043	p_1–2_ = 0.5 p_1–3_ = 0.144 p_1–4_ = 0.225	

CPB—cardiopulmonary bypass.

**Table 10:** Glomerular filtration rate and urine output in the studied groups (mL/min), median (Q1; Q3), *n* = 10.

Point	CPB-contr, *n* = 5	CPB-NO, *n* = 5	Mann–Whitney U-Test
Baseline (1)	81.7 (79.2; 86.5)	83.6 (81.5; 83.9)	*p* = 1.00
Weaning CPB (2)	73.3 (68.5; 76.6)	83.5 (80.2; 84.5)	*p* = 0.548
6 h after CPB (3)	67.9 (62.3; 69.2)	78.9 (77.8; 82.3)	*p* = 0.016
12 h after CPB (4)	50.3 (48.7; 54.9) (0.042)	67.7 (65.5; 68.0)	*p* = 0.032
Wilcoxon test	p_1–2_ = 0.008 p_1–3_ = 0.043 p_1–4_ = 0.042	p_1–2_ = 0.5 p_1–3_ = 0.144 p_1–4_ = 0.043	
Urine output in operation procedure mL/kg/h	2 (1.75; 2.55)	4.5 (4; 4.5)	*p* = 0.015
Urine output in postoperative period mL/kg/h	1.67 (1.35; 2.19)	2.09 (1.67; 2.29)	*p* = 0.53

CPB—cardiopulmonary bypass.

**Table 11:** Plasma free hemoglobin levels in the studied groups (g/l), median (Q1; Q3), *n* = 10.

Point	CPB-contr, *n* = 5	CPB-NO, *n* = 5	Mann–Whitney U-Test
Baseline	0 (0; 0)	0 (0; 0.2)	*p* = 0.134
Weaning CPB	0.2 (0.1; 0.2)	0 (0; 0)	*p* = 0.106
6 h after CPB	0 (0; 0)	0 (0; 0)	*p* = 1.000
12 h after CPB	0 (0; 0)	0 (0; 0)	*p* = 0.317

CPB—cardiopulmonary bypass.

**Table 12:** NGAL levels in the studied groups (ng/mL), median (Q1; Q3), *n* = 10.

Point	CPB-contr, *n* = 5	CPB-NO, *n* = 5	Mann–Whitney U-Test
Baseline (1)	62.1 (48.3; 92.8)	69.4 (43.3; 140.6)	*p* = 0.77
Weaning CPB (2)	63.6 (48.2; 234.4)	96.7 (84.3; 140.1)	*p* = 0.6
6 h after CPB (3)	95.9 (64; 101.7)	57 (41.8; 87.8)	*p* = 0.22
12 h after CPB (4)	287.9 (100.1; 296.3)	102 (53.2; 150.9)	*p* = 0.14
Wilcoxon test	p_1–2_ = 0.686 p_1–3_ = 0.345 p_1–4_ = 0.043	p_1–2_ = 0.715 p_1–3_ = 0.144 p_1–4_ = 1.000	

CPB—cardiopulmonary bypass.

**Table 13:** Parameters of histological changes in kidney tissue of animals of the studied groups, scores—median (Q1; Q3).

Value	Group	Value, Point	Mann–Whitney U-Test
Dystrophy of the epithelium of the tubules	CPB-contr, *n* = 25	2 (1; 3)	*p* = 0.49
	CPB-NO, *n* = 25	1 (1; 2)	
Lymphoplasmocytic infiltration	CPB-contr, *n* = 25	0 (0; 1)	*p* = 0.37
	CPB-NO, *n* = 25	1 (1; 1)	
Hyperemia	CPB-contr, *n* = 25	2 (2; 3)	*p* = 0.55
	CPB-NO, *n* = 25	3 (2; 3)	

## Data Availability

Not applicable.
